# The politics of reproduction and the realities of obstetric violence in Ghana

**DOI:** 10.3389/fgwh.2025.1627928

**Published:** 2025-09-22

**Authors:** Abena Asefuaba Yalley

**Affiliations:** 1 Department of Politics and Public Administration, University of Konstanz, Konstanz, Germany; 2 Zukunftskolleg, University of Konstanz, Konstanz, Germany

**Keywords:** women, childbirth, obstetric violence, abuse, Ghana

## Abstract

**Introduction:**

Violence during childbirth, widely conceptualized as obstetric violence, is a precarious and pressing public health concern. These include brutal acts of physical violence, humiliation, forced medical care, as well as denial of treatment. The World Health Organization recognizes it as torturous acts that put the lives of many women at risk. This paper explores the dynamics of obstetric violence through the birth narratives of women in rural and urban Ghana.

**Methods:**

Qualitative phenomenological research was conducted in eight rural and urban public health facilities in the Western and Ashanti Regions of Ghana. A total of 35 women (20 from urban areas and 15 from rural areas) who had given birth in the last 24 months at health facilities were purposively selected and interviewed between August 2021 and February 2022 using a semi-structured interview guide. Thematic data analysis was conducted using the NVivo qualitative data analysis software.

**Results:**

The findings of the study revealed that there is a pervasive culture of violence surrounding childbirth, with women describing their childbirth memories with sadness and regret. Obstetric violence manifests in the form of physical violence, where sutures after episiotomies are performed without anesthesia, and women are beaten or slapped for their inability to push. In addition, women are grossly abandoned, usually during the second stage of labor. Sometimes, the entire care is halted when healthcare workers are provoked or feel that the women do not make enough efforts. In some cases, women are even left to deliver unassisted. Yelling, shouting, and verbal abuse of women were very dominant, and this particularly instilled fear in women, which prevented them from seeking help in critical situations, thereby increasing the risk of birth complications. Teenage mothers and HIV-positive women are predominantly discriminated against. Psychological trauma, mistrust in health institutions, and preference for unskilled birth attendants are the major consequences of obstetric violence.

**Discussion:**

Overall, obstetric violence is a major setback in Ghana's effort to achieve the global target of reduced maternal mortality. There is a critical need for the Ghanaian government to develop interventions to tackle this challenge.

## 1 Introduction

Historically, childbirth was in the exclusive domain of traditional midwives (1). These midwifery skills were passed down from generation to generation, with a great emphasis on the physiological aspects of birthing (1, 2). However, with the evolution of Western medicine and the imposition of colonial rule in many countries, including Ghana, indigenous medical knowledge and traditional midwifery were replaced by mainstream medicine (3, 4). Today, the greater proportion of births worldwide are hospitalized, with considerable emphasis on the biomedical model of care. In Ghana, up to 75% of deliveries occur in healthcare facilities (5, 6). Birthing is now perceived as a pathological process that demands radical medical intervention. This also comes with a loss of autonomy for women with regard to their bodies and being subjected to established social norms and practices (7). While global trends on maternal mortality have reduced, recent investigations into women's childbirth experiences have disclosed shocking revelations of abuse and mistreatment associated with institutional birthing (8–10). These abuses, widely conceptualized as obstetric violence, have received widespread attention as a pressing public health concern. Obstetric violence is the violence exercised by healthcare personnel on the body and reproductive processes of women and manifests in dehumanizing treatment, over medicalization, and authoritative care (11). They include situations where women are sometimes physically abused, disrespected, verbally abused, or even abandoned to deliver without any professional assistance. Although obstetric violence is not a new phenomenon, women only began to break the silence in the mid-2000s through the Humanization of Childbirth Movement in Latin America. This is partly due to the pervasive societal acceptance of violence against women, prompting many women to live in silence in the face of abuse in healthcare institutions. One of the earliest works on obstetric violence by Bowser and Hill (12) on the global outlook of obstetric violence classified it into seven categories: “physical abuse”, “non-dignified care”, “non-consented care”, “discriminated care”, “non-confidential care”, “neglected care”, “detention in the health facility”. The World Health Organization, recognizing its widespread and systemic nature, released a statement on the prevention and elimination of abuse during facility-based childbirth, calling for more research into the phenomenon (10).

Obstetric violence can impact the health and well-being of the mother and the new-born in both direct and indirect ways (13). Mistreatment during childbirth can lead to physical harm and medical complications (14). It can also negatively impact breastfeeding and maternal-infant bonding (15), and reduce future healthcare engagement, putting both mother and child at higher risk for untreated health conditions (16). Women who have experienced obstetric violence are mostly vulnerable to mental health disorders, particularly postpartum depression and Post-Traumatic Stress Disorder. Recent studies have revealed a close correlation between obstetric violence and postpartum depression and increased post-traumatic stress disorder (PTSD) (17), making quality obstetric care imperative for women.

A systematic review of the global prevalence of obstetric violence indicated that 52% of women experience at least one form of abuse during pregnancy, childbirth, or during the postnatal period (18). Research in high-income countries has discovered a prevailing rate of 77% in Germany (18), 81% in Poland (19), 67% in Spain (20), 17.2% in the US (21), and 45% in the Netherlands. In Africa, studies have also reported high prevalence of obstetric violence associated with facility-based deliveries. Bohren et al. (16) established that the overall magnitude of obstetric violence in four African countries was 41% with 14% of women experiencing physical violence, 37% reporting verbal abuses, and 11% facing stigma and discrimination. In addition, 10% of the cesarean sections conducted were without the consent of the women. Findings on individual countries indicated 67.7% in Nigeria, 39% in Guinea, and 77% in Ethiopia (16, 22).

In Ghana, the prevalence of obstetric violence ranges between 65% and 83% (23, 24). Yalley et al.'s (25) most recent study revealed that two in every three Ghanaian women who utilized health facilities experienced abuse during childbirth, with rural women experiencing more physical violence. This high prevalence makes obstetric violence a general societal problem, with long-lasting consequences on women's mental and physical health (26). The mistreatment and violence that women experience in reproductive health services is not detached from the broader context of structural inequality, discrimination, and patriarchy (27). Absence of appropriate education for women alongside the ignorance of women's rights can also fuel this form of violence (28). In addition, the power dynamics between healthcare professionals and care receivers combine with societal prejudices to put women in a hugely vulnerable position for abuse. Healthcare professionals have acknowledged obstetric violence as widespread in Ghana and have associated it with poor working conditions in the healthcare system (23, 29). The pervasive nature of obstetric violence in Ghana puts the lives of many women at risk. Poor quality of care in the process of labour, with neglect being the most common, is closely linked to the preventable maternal mortality rate (30). Ghana's maternal mortality is still far above the global target (70 deaths in every 100,000 births), with 263 Ghanaian women dying in every 100,000 live births (31). Improving the quality of obstetric care is significant in reducing these numbers. Although obstetric violence is a pressing public health concern, its dynamics have still not been thoroughly investigated in Ghana, particularly in the rural areas where maternal mortality is most predominant (32, 33). Understanding the dynamics and manifestations of obstetric violence is key to designing contextually appropriate interventions to mitigate it. This study, therefore, explores the experiences of obstetric violence and the perceived impacts among rural and urban women in the Ashanti and Western Regions of Ghana.

## 2 Materials and methods

### 2.1 Study setting and design

The study adopted the qualitative phenomenological research approach to explore the dynamics of women's experiences of obstetric violence. Qualitative phenomenology is particularly appropriate for exploring a specific phenomenon thoroughly and for acquiring comprehensive information regarding personal experiences and perspectives (34), offering a critical understanding of the peculiarities and manifestations of obstetric violence in Ghana. The study was conducted in eight health facilities in the Western and Ashanti Regions of Ghana. The health facilities were selected if they were public institutions, provided obstetric care services to women, and had a high client flow for maternity services. In the Ashanti Region, data was collected in the Maternal and Child Hospital and the Tafo Government Hospital (located in the Kumasi Metropolis), and Nkenkaasu Government Hospital and Ejura District Hospital (serving the rural communities). Empirical data in the Western Region was collected in the Kwesimintsim Polyclinic and Essikado Government Hospital (urban) and the Agona Nkwanta Health Center and Dixcove Government Hospital located in the rural part of the Western Region.

### 2.2 Study population and sampling

The study population comprised women who had given birth at the selected health facilities in the past 24 months. A total of 35 women were purposely selected and interviewed using a semi-structured interview guide. Women who delivered outside the selected health facilities and women who lost their babies at childbirth were excluded from the study. Overall, the majority of the women (20) had given birth in the urban health facilities, while 15 had utilized the rural health facilities for childbirth. The principle of saturation in qualitative research guided the selection of the sample size. It is a research technique whereby the researcher ceases collecting data when subsequent interviews reveal no new information (35). Hence, data collecting stopped at the point of saturation, making the study sample size sufficient for interrogating the dynamics of obstetric violence in Ghana.

### 2.3 Data collection procedure

Prior to the recruitment of the women, the researchers met with the hospital administrators, where the rationale of the study and the ethical approval letter were presented for their consent. The principal investigator recruited and trained two research assistants (one for each region) to conduct the interviews with the women. The female research assistants were non-clinical staff with a bachelor's degree and extensive experience in collecting qualitative interviews. The training included the research protocol, sampling procedure, ethical issues in researching violence against women, and qualitative interview techniques. The recruitment of the women was conducted at the child immunization clinics situated in the respective health facilities. The researchers approached the women at the clinics and provided them with an adequate explanation of the study's rationale. Women who met the selection criteria were invited to participate in the study, and those who consented were scheduled and interviewed in their preferred locations. The interviews explored women's childbirth experiences in the health facilities with particular emphasis on their experiences of obstetric violence, witnessing violence in the labor ward, the perceived impact of obstetric violence on women, as well as the sociodemographic information of the participants. Obstetric violence was measured based on the seven performance indicators developed by Bowser and Hill (12). The interviews were conducted in Akan, and they were audio recorded with the consent of the participants. The interviews were conducted between August 2021 and February 2022.

### 2.4 Data analysis

All the recorded interviews were transcribed and translated into English, and the data analysis was performed based on Braun and Clarke's (36) framework for conducting thematic analysis. The framework entails the identification of emerging themes and patterns from the qualitative data in order to answer the research questions. At the first stage, the interviews were read meticulously for familiarity and checked for inconsistencies. At the second stage, the data was transferred to NVivo Qualitative Data Analysis Software, Version 12. Thereafter, codes and sub-codes were generated using the inductive approach, which involved repetitive reading of the data for the identification of common or emerging themes. For the purpose of this paper, only codes that focused on women's experiences of obstetric violence were extracted and analyzed. The identified codes were furthermore reviewed for major themes and sub-themes, which were validated by the research assistants. The rigor of the research was ensured through debriefing, transferability, reflexivity, and audit trail. The utilization of the purposive sampling technique ensured that only participants with the required knowledge and experience were recruited and interviewed. Also, the principal investigator held constant meetings with the research assistants for debriefing. To promote transferability, the protocol of the study was strictly followed, while a thorough description of the methodology of the study allows for a future replication.

### 2.5 Ethical considerations

The research was approved by the Ghana Health Service Ethics Review Committee (GHS-ERC 010/06/21) and the Ethics Committee of the University of Konstanz, Germany (IRB Statement 37/2021). In addition, the consent of all participants was sought before participation. Obstetric violence is a sensitive topic which requires strong confidentiality and anonymity. To safeguard the confidentiality of participants, their names and contact details were not collected.

## 3 Results

This study explored women's experiences of obstetric violence during childbirth. Six main categories of obstetric violence emerged from the data: physical violence, abandonment, humiliation, discrimination, denial of birth companions and preferred birth positions. The major themes regarding the perceived impact of obstetric violence were: physical health, psychological trauma, distrust in healthcare providers and preference for unskilled birth attendants.

### 3.1 Physical violence

Physical abuse of women in labor was one of the major themes that emerged from the childbirth narratives. Laboring women were subjected to incessant brutalities by healthcare professionals for their inability to push or follow instructions. Several women admitted they were beaten, slapped on the thighs or the face during childbirth. Other torturous acts of violence include stitching women without anesthesia, usually after episiotomies or tears, which women described as unbearable. Women admitted that there is a general normalization of violence in the delivery rooms, perceiving it as part of the childbirth process. The following narratives from the interviews portrayed the realities of physical violence in the delivery rooms:

The experience I had during my delivery was very bad. I bled a lot to the point that I collapsed. The first doctor who stitched me inserted his hand in my vagina to stitch it up without giving me any anesthesia, and I was bleeding on top of it. I really suffered, so it made me decide not to go there again. (Mother 15)

While I was pushing, I became tired and stopped pushing. The midwife slapped my thighs while I was pushing, but I think it happens to everyone. (Mother 2)

Another form of physical violence involved the intentional blocking of women's breath during the second stage of delivery. Healthcare professionals inflict pain on women by forcefully holding their nostrils and mouths to demonstrate the state of the baby passing through the birth canal. This strategy was particularly used to stimulate women to push. A woman giving an account of what she witnessed at the labour ward revealed that:

The midwives told one woman to push, but she was tired and could not push. The midwives then closed her mouth and held her nose, and they asked her if she could breathe and she said no. Then they told her that is how the baby is feeling, so she should push. (Mother, 25)

There were also cases of multiple abuses- physical violence followed by other forms of violence, mainly abandonment. This was often inflicted on women who fought back or refused to accept mistreatment. Violence was employed as a tool of punishment, to keep women under the authority of medical professionals.

There's this girl that the midwives beat mercilessly, and they told her they will transfer her to Techiman if she doesn't keep quiet. The girl also insulted them. They got angry and left her. If it hadn't been for me, her baby would have died. They left her, ignored her and went to sit down conversing. When she began shouting that the baby was coming, they didn't mind her. (Mother 14)

From the above narratives, it is apparent that physical violence during childbirth is common, making the entire atmosphere stressful for the laboring woman.

### 3.2 Abandonment

Childbirth requires continuous monitoring of women and the child even after postpartum. However, the experiences of the women interviewed depicted a rather gross neglect or abandonment. Although the abandonment of women could occur during all three stages of childbirth, the most common occurs during the second stage of delivery, where women need crucial care. Sometimes, the entire care is even halted when healthcare professionals are either provoked or feel women are not making enough efforts. A mother revealed that the midwife stitching her stopped the entire process because the woman accidentally splashed water on the midwife. The following testimonies of women reveal the ubiquitous nature of abandonment in healthcare facilities:

There was one nurse who kept coming to examine me from time to time. The last time she came, the baby was coming, and I couldn't do anything. She asked me to lie down so that she could examine me and I told her that the way I'm feeling around my abdomen, I can't climb the bed. She got angry and went to stand somewhere murmuring. After a while, I felt the baby's head between my thighs, so I called her. She told me to get away and that if she is not done with whatever she is doing, she won't come. (Mother 10)

When I realized that the baby was about to come, I shouted to call them [midwives], but no one answered me. Then one of the mothers in the labor ward said that if the head is coming, I should just go ahead and push; otherwise, I will lose the baby. So, I pushed the baby before they later came. Because of this, I have decided that I won't give birth at a government hospital again because I don't want to go there and be humiliated. (Mother 19)

Some of the things that make me sad are how I was neglected when it was time for me to give birth. From the contractions, I could tell labor had started, but the midwives kept saying labor had not started without conducting any proper examination. The midwife refused to attend to my needs even after my water broke. All these make me sad now that I think about them, but I'm glad that I've finally been discharged. (Mother 13)

There are also instances where women are abandoned and left to deliver without any assistance from health care professionals. Women describe their experiences as horrific, dehumanizing, and torturous. A woman, narrating her experience, disclosed that:

After I was fully dilated, the nurse kept saying “push, push”. Then she took a chair and sat on it far away from me. I believe that if she were standing close to me, I wouldn't have had any tears. When she saw that the baby had come out with so much force at once and was almost falling, she got up quickly from the chair to catch the child to prevent it from falling. So, I got the tear because of this process. (Mother 28)

This was corroborated by another woman:

I told the midwife that my baby's time is due, but she said the time wasn't up. But I insisted that the baby was coming because I could feel the head down there. Then the baby came out before the midwife came. (Mother 34)

### 3.3 Humiliation

The women described the enormous humiliation that they received from healthcare professionals. Several women admitted that they were yelled at, verbally abused, openly mocked, and disrespected. Words such as “nasty” and “disgust” were used by health workers to describe women. The women admitted these treatments are completely dehumanizing and demeaning. Particularly, shouting (which is the most common) instilled fear in women, which sometimes prevents them from asking for help in critical situations. Humiliation is therefore a way of silencing women, making them more vulnerable to birth complications.

Sometimes they [midwives] can even ignore you and they will be pressing their phones. The way they talk to you is as if they are talking to a child. When I pushed and the child was coming, I went to toilet in the process, and the midwife told me that she cannot clean any nasty thing over here. (Mother 12)

Another woman, describing how she was insulted, noted that:

The doctor who came to dress me up is the one who gave me some problems, and it really made me sad. When I'm pregnant, I spit a lot and because of this, I always chew a stick. Before the surgery, they told me not to eat or drink, so I put the stick into my mouth since my throat would become dry if I kept spitting saliva. The doctor asked me who permitted me to chew the stick, and I told him everything. Then he started insulting me. He said the stick is food. He insulted me so well, and I could tell that the dressing he was doing for me was being done with some kind of anger and disgust. I became frightened. (Mother 30)

Although women have adequate knowledge about their reproductive health and experiences, practitioners often perceive women as ignorant. Healthcare professionals act on this preconceived notion of ignorance and disrespect them, demeaning their decisions and choices regarding contraceptives.

When I went to the hospital, the midwife started saying, “Ei Maame [woman], when do you and your husband want to stop giving birth?” She really humiliated me. For me, family planning doesn't help me. Any time I do it, I have lots of complications. I even got asthma. When I took the injection for the first time, it gave me consistent asthmatic attacks. Also, anytime I take the tablets, I have issues with my leg. Because of this, one day I took their leaflets [contraceptives] and read them, and I saw that the disadvantages are greater. (Mother 6)

When I gave birth to my third child, it wasn't long before I got pregnant again. When I went to the hospital, one woman was heavily bleeding and shouting that her head was aching terribly. Then the nurse started shouting [at me], “Wasn't it recently you came to give birth and you're here again? See your colleague [referring to the woman bleeding] there bleeding heavily”. I don't want to go there again to be humiliated. (Mother 30)

A greater number of women were displeased and worried about the disrespect and shouting that pervaded the healthcare services for women. The hostility of healthcare workers is undeniably toxic for laboring women.

Some people [health providers] don't know how to talk. They look at you anyhow; even when they are supposed to take care of you, they will shout at you. Do you understand? (Mother 19)

As for Nkenkaasu here, everyone knows that the health workers like shouting at us and mistreating us. We always mention their names, but you can't deprive someone of his source of income. (Mother 8)

I get scared sometimes! I just pray to meet a nice person the next time I go to the hospital to deliver my baby. There was a nurse who would shout at us for the slightest mistake we made. It was so bad that we were even scared to tell her our complaints. (Mother 32)

### 3.4 Discrimination

The women interviewed confessed that one of the major problems they had to face during childbirth was discrimination. Women have the right to receive good and equal level of care. Beyond this, the ethical principle of care demands that everyone is provided with equal care irrespective of their socio-economic, religious, educational, or ethnic background. However, women identified preferential treatments for healthcare workers who are pregnant. For example pregnant doctors, midwives, nurses and health administrators are treated with much respect. Also, the women knew of special facilities that are reserved only for healthcare workers. Some women recounted:

All I can say is that we should do to others what you want others to do to us. Because this is a life and death matter. The way the midwives attend to their colleagues is different from the way they attend to us. But we are all the same; therefore, they have to treat all of us equally. And if you treat people well, God will bless you. (Mother 1)

Oh, there is a place called a side ward. If you are a nurse [or midwife] and in labor, you will be sent to the side ward. There is also the main ward, which is quite big. That is the place for the public. In the side ward, there are only four beds. (Mother 12)

Sometimes, some midwives declare their stance on the type of care they will provide for women from a poor socio-economic background. Women's appearance determines the kind of care they will receive.

One day, the midwife said that if you [woman] come to the hospital and you are not well kept- you don't wear sandals, but you are dirty, your hair and the dress you are wearing are not kept well -she [midwife] will not attend to you. Even when you are in labor, she will not take it easy with you. She says that all the time. (Mother 26)

Discriminatory care was not only based on women's socio-economic background, but also their medical status. In this study, women who are HIV positive are discriminated against and receive poor care. An HIV positive mother narrated that:

I was treated well until the day of delivery. I was told I had a second-stage delay, but I could feel the baby's head, and when the midwives examined me, it was confirmed I was ready for delivery. I expected that they would help me to get to the labor room in a wheelchair; instead, they asked me to walk there all by myself. I had been lying in one position for a very long time and so I could not move my leg. I asked for help again, and they still refused to help. So I had to manage to walk to the labor room all by myself, and I fell. I pleaded again with them to help me get up, but they still didn't help. The reason being that they didn't want to be infected with HIV. Those were sad memories that I never want to think about. I was maltreated because I was HIV positive. I usually don't worry so much about my HIV status, but on that day, I thought so much about it and it made me sad. I thought to myself that I would have been treated better had it not been for my health condition. (Mother 7)

There was a consensus that teenage mothers are severely abused and mistreated. One of the mothers, describing what she witnessed in the labor ward, revealed that:

Maltreating underage pregnant women is a common thing. It was her first time delivering a baby, so she didn't have any experience with how to push, and so couldn't tolerate the pain. That's why she was shouting in pain, but they maltreated her. (Mother 11)

Another woman emphasized that:

I saw it. When you are very young, they will try to intimidate you. Maybe you are 16 years old, say a teenager. (Mother 3)

### 3.5 Denial of birth companion and preferred birth position

The right to determine and select a preferred birth position is a fundamental right of every laboring woman. The findings of this study, however, revealed that desired birth position is not a choice for many women. Health workers only insist on one position for all women –the lithotomy position. A woman noted, “I was not given options to choose. I was just made to lie down”. One woman also pointed out that “even when you want to get off the bed, they keep asking you to lie back on the bed”. Apart from the restrictions on birth positions, women are also not allowed to have birth companions. Many women expressed their need for birth companions, particularly their partners, to provide support. They also emphasized that having their husbands in the delivery rooms helps evoke compassion, respect, and better treatment. While there are concerns of privacy for other women due to infrastructural deficits, in some cases, birth companions are deemed unnecessary by healthcare professionals.

No relatives were allowed in there. The midwives made it clear that their job was to help me with whatever I needed, so there was no need for someone else to be with me. They said they will take care of me themselves. (Mother 8)

If my husband is around, at least he will give me some comfort, but they don't allow it here. We see it on television, but here, when you come to the hospital, the way they will treat your husband, you will not even like it. (Mother 9)

Relatives, especially husbands, should be allowed into the labor rooms. They should be allowed in there so they can witness the pain women go through during labor. If they witness it, they'll treat women with respect. (Mother 10)

Undeniably, birth companions are crucial for positive birth experiences, but women are denied this opportunity.

### 3.6 Perceived impact

Obstetric violence had diverse impacts on women's wellbeing. These include negative consequences on their physical health, psychological trauma, mistrust in health institutions, and preference for unskilled birth attendants.

#### 3.6.1 Physical health

Some of the women revealed that their experiences of obstetric violence had negative health consequences, which included excessive bleeding, preventable tears, and health challenges for their babies.

It affected my baby. She is unable to use her left leg the way she is supposed to till now. Recently, I took her to the hospital. I was told she has a problem with her eyes also. I think that is a result of the delay during the second stage of labor. (Mother 7)

I became frightened and I began to bleed. So, they [new doctors] had to undo the stitches and redo the stitches before the bleeding stopped. (Mother, 15)

#### 3.6.2 Psychological impacts

The narratives of the women portrayed an aura of discomfort, negative emotions, and regrets associated with institutional births. The women also felt insecure and helpless.

Anytime I go there [the hospital], I'm unhappy. If I had my family here, I wouldn't be going there. Because sometimes even if you [the woman] have a problem, you cannot tell them [healthcare workers] because you don't know what response you will get. (Mother 26)

Sometimes when women go to the hospital, the midwives even ignore them. And when it happens like that and there is a problem, they [women] don't even want to tell the midwife. The second time that I came [to the hospital], I was heartbroken. It made me think a lot. (Mother 35)

It made me feel bad and made me regret coming there [the hospital]. So, I was telling my husband the other time that if I ever give birth again, I won't go to a government hospital again. Because I don't want to go there and be humiliated. (Mother 30)

#### 3.6.3 Mistrust in healthcare institutions

The women also raised concerns that obstetric violence reduces women's trust in healthcare workers and the entire health system. This prevents women from sharing their problems and health concerns with healthcare professionals.

You know that childbirth itself is like life and death! There are some women who, even the way you [midwife] talk to them [in a calm manner], it gives them hope, and they have trust in the system. But once a midwife shouts at them, it puts fear in them, and during that process, the person can even die. But if you [the midwife] come down to their level, talk to them, and encourage them, they will know that you are giving them hope, you know what you are doing, so they will trust you. But when you [midwife] are shouting, the woman becomes confused. (Mother 17)

When women have issues at home and they go to the hospital, they have hope that their issues will be solved there. But you [the woman] will go and they [healthcare workers] will be shouting and maltreating you [the woman]. So, the shouting and the other things [maltreatment] don't allow women to tell the nurses their problems. (Mother 16)

#### 3.6.4 Preference for unskilled birth attendants

While some women strongly affirmed a decision not to reuse public health facilities for childbirth, many pointed out their decision to use unskilled birth attendants in the future. Some women strongly opined that obstetric violence is one of the major reasons for women's preference for unskilled birth attendants, although the risks of death in cases of abrupt complications are high.

I was really hurt and disturbed. Oh, if labor had set in while I was at home, I would have given birth there. As for the hospital, I've told myself that if I get pregnant again, I won't give birth in the hospital again. (Mother 27)

It is because of such behaviors [mistreatment] that they [women] become scared, so they mostly want to give birth at home. And giving birth at home, too, a lot of women die. (Mother 5)

I'm never attending this hospital for labor anymore after all that has happened. I can attend ante-natal care here, but I'll never deliver my baby in this hospital again. (Mother 4)

## 4 Discussion

This study explored the dynamics of obstetric violence in Ghana through women's narratives. The study revealed the ubiquitous nature of obstetric violence in healthcare facilities. This is consistent with quantitative studies on obstetric violence in Ghana, where a high prevalence of 65% was reported (23). The prevailing nature of obstetric violence has been acknowledged by healthcare practitioners who admit witnessing and perpetrating these acts of mistreatment (23, 37, 38). In this study, obstetric violence manifested in the form of physical abuse, neglect or abandonment of women, humiliation, discrimination, and denial of birth companions and preferred birth positions. The consequences of obstetric violence included physical and psychological health impacts, mistrust in healthcare systems, and impediments to women's utilization of skilled birth attendants in Ghana. These clearly demonstrate that obstetric violence is a major setback in Ghana's maternal healthcare, demanding critical actions.

Regarding physical violence, women admitted being beaten and slapped while sutures following episiotomies were stitched without anesthesia. In other instances, healthcare practitioners intentionally blocked women's nostrils to compel them to push. Comparing these findings to the literature, Yalley et al.'s (23) study indicated 27% of Ghanaian women experience physical violence during childbirth, with the vast proportion (12%) being stitches of episiotomies without anesthesia. Similar findings have been documented in India, where episiotomy repairs without anesthesia were found to be a common practice in many healthcare facilities (39). The stitching of the perineum without anesthesia is considered human torture, cruel, and a treacherous human rights violation (13, 40). This study reflects a culture of punitive care and noncompliance with ethical standards of care in Ghana, exacerbated by an overstretched health system and poor training. The health workforce in Ghana is plagued by skill and knowledge gaps, which undermine quality care for women (41). Increased training and professional oversight on the conduct of healthcare practitioners is key to dealing with obstetric violence.

Abandonment of women emerged as a notable form of abuse, similar to Nigeria, Ethiopia, Kenya, Brazil, Argentina, and Germany (15, 17, 42–44). Although this is inevitably a global issue, in the Ghanaian context, the power dynamics stemming from an authoritative culture over women and unprofessionalism underpin it. In literature, abandonment has often been associated with workload and poor working conditions as well as limited facilities (39, 45, 46). In this study, however, it was often a deliberate act to punish or to subjugate women. The pervasive professional misconduct is the manifestation of an authoritative health system where women are abused with impunity. This disempowered women within the healthcare system, subjecting them to unacceptable violence and humiliation. Unquestionably, obstetric violence is a gendered phenomenon deeply rooted in gender ideals about women and their position in society. Collins et al. (17) contend that the power imbalance in healthcare is a reflection of a patriarchal system designed to keep women under authority. Structural changes that deal with stereotypical gender norms and other sociocultural factors are crucial to tackling obstetric violence in Ghana. Moreover, prominent action in enforcing legal frameworks for maternal care and actions against obstetric violence is imperative.

Humiliation or non-dignified care manifested in the form of outright verbal abuse, mocking, yelling, and total disrespect for women, including their decisions, thereby instilling fear and creating a hostile and toxic birthing environment. Global mapping of women's experiences of obstetric violence revealed similar patterns of dehumanized care (12, 18). In Australia, one in ten women described feeling dehumanized, violated and powerless in the delivery room (47). These actions compound to reduce the quality of childbirth experiences, which experts argue are crucial for wholesome maternal health (48, 49). In the context of obstetric care in Ghana, these treatments, amplified by the prevailing shouting used by healthcare professionals in the labor wards, reduce women's voice and autonomy in a hierarchical medical system. In addition, the vulnerable position of pregnant women could deter them from demanding dignified care. This is even more complex when women are not allowed access to birth companions, which is crucial to reducing abuses during deliveries (38, 50). There were cases of discrimination, particularly against teenage mothers and women with HIV, revealing how diverse layers of vulnerability increase the propensity for obstetric violence.

Obstetric violence had dire consequences on the physical and mental health of women. Intense pain and other health complications due to neglected care were reported. While it is uncertain whether these physical health challenges are a direct consequence of obstetric violence, the women were highly convinced of their experiences and the repercussions on their health. A study in India found a strong association between obstetric violence experiences and complications during childbirth and in the postpartum period (14). In this study, psychological trauma following experiences of abuse and mistreatment was profound in the narratives of women. These included sadness, regrets, heartbreaks, and negative emotions. Experts contend that “psychological trauma can leave you struggling with upsetting emotions, memories, and anxiety that will not go away, impacting the sense of security, and resulting in the feeling of helplessness” (51). The contributing factors of traumatic births include “poor relationships, limited decision making, inadequate support, and over-medicalization” (17). Worldwide, one in three women describe their childbirth experiences as traumatic, with 6%–10% likely to experience PTSD (52, 53). A cross-sectional study in the Netherlands established that the lack of autonomy in decision making is linked to trauma in 30% of women (54). This can affect women's ability to bond with their babies, breastfeed, and hamper their quality of life, including their ability to trust people (17). In this study, obstetric violence violated the trust that women had in the healthcare providers, leading to a preference for unskilled birth attendants.

## 5 Conclusion

This study explored the dynamics of obstetric violence in the Ashanti and Western Regions of Ghana. The study highlights the ubiquitous nature of obstetric violence and the impact it has on women. Women experienced six forms of abuse: physical violence, abandonment, humiliation, discrimination and denial of birth companions and birth position of choice. Although the experience of obstetric violence was common to all women, the mistreatment of teenage mothers is the most profound. Obstetric violence greatly affected the psychological well-being of women, leading to mistrust in the healthcare system and preference for unskilled birth attendants. The study points to the need for an intervention to deal with the problem of abuse during childbirth. As highlighted in the study, there is a critical need for the training of healthcare workers on respectful maternity, while oversight or monitoring in the labor wards could increase health workers' accountability. It is also imperative to deal with structural changes, particularly gendered norms and stereotypes that facilitate the perpetration of obstetric violence. Also, there is a need for women to be empowered more on their reproductive rights, particularly during labor. Other systemic changes, such as gender sensitive wards, are key for maternity care to safeguard the privacy of women and promote birth companionship, which are crucial to reducing obstetric violence.

## 6 Strengths and limitations

This study provides a detailed account of birthing women's experiences in Ghana, providing a robust understanding of the manifestations of obstetric violence. The in-depth interviews highlighted critical experiences which could not be captured with numbers. Nonetheless, the qualitative nature of the study limits its generalization due to the small sample size. More so, the recruitment of women in the health facilities could lead to social desirability as women may underreport their experiences. Nonetheless, obstetric violence was profound in women's narratives. This study is also unlikely to be affected by recall bias as research has established that childbirth memories can last until 20 years (55).

## Data Availability

The raw data supporting the conclusions of this article will be made available by the authors, without undue reservation.
